# Assessment of the effectiveness of physical activity interventions in the Brazilian Unified Health System

**DOI:** 10.1590/S1518-8787.2017051006654

**Published:** 2017-06-20

**Authors:** Evelyn Helena Corgosinho Ribeiro, Leandro Martin Totaro Garcia, Emanuel Péricles Salvador, Evelyn Fabiana Costa, Douglas Roque Andrade, Maria do Rosario Dias de Oliveira Latorre, Alex Antonio Florindo

**Affiliations:** I Programa de Pós-Graduação em Nutrição em Saúde Pública. Faculdade de Saúde Pública. Universidade de São Paulo. São Paulo, SP, Brasil; II Grupo de Estudos e Pesquisas Epidemiológicas em Atividade Física e Saúde. Universidade de São Paulo. São Paulo, SP, Brasil; III Departamento de Educação Física. Universidade Federal do Maranhão. São Luís, MA, Brasil; IV Escola de Artes, Ciências e Humanidades. Universidade de São Paulo. São Paulo, SP, Brasil; V Departamento de Epidemiologia. Faculdade de Saúde Pública. Universidade de São Paulo. São Paulo, SP, Brasil

**Keywords:** Adult, Motor Activity, Outcome Assessment (Health Care), Health Behavior, Health Promotion, Unified Health System, Randomized Controlled Trial

## Abstract

**OBJECTIVE:**

To assess the effect of interventions on the levels of physical activity of healthy adults, users of the Brazilian Unified Health System and attended by the Family Health Strategy.

**METHODS:**

Non-randomized experimental study with 157 adults allocated in three groups: 1) physical exercise classes (n = 54), 2) health education (n = 54), 3) control (n = 49). The study lasted for18 months, with 12 months of interventions and six months of follow-up after intervention. Assessments took place at the beginning, in the 12 months, and in the 18 months of study. Physical activity has been assessed by questionnaires and accelerometry. For the analyses, we have used the intention-to-treat principle and generalized estimating equations.

**RESULTS:**

After 12 months, both intervention groups have increased the minutes of weekly leisure time physical activity and annual scores of physical exercise, leisure and transport-related physical activity. The exercise class group has obtained the highest average annual physical exercises score when compared to the other groups (p < 0.001). In the follow-up period, the exercise class group reduced its annual score (average: -0.3; 95%CI -0.5–-0.1), while the health education group increased this score (average: 0.2; 95%CI 0.1–0.4). There have been no differences in the levels of physical activity measured by accelerometry.

**CONCLUSIONS:**

The interventions have been effective in increasing the practice of physical activity. However, we have observed that the health education intervention was more effective for maintaining the practice of physical activity in the period after intervention. We recommend the use of both interventions to promote physical activity in the Brazilian Unified Health System, according to the local reality of professionals, facilities, and team objectives.

## RESUMO

**OBJETIVO:**

Avaliar o efeito de intervenções nos níveis de atividade física de adultos saudáveis, usuários do Sistema Único de Saúde e atendidos pela Estratégia de Saúde da Família.

**MÉTODOS:**

Estudo experimental, não randomizado, com 157 adultos alocados em três grupos: 1) classes de exercícios físicos (n = 54); 2) educação em saúde (n = 54); 3) controle (n = 49). O estudo teve duração de 18 meses, sendo 12 meses de intervenções e seis meses de acompanhamento pós-intervenção. As avaliações ocorreram no início, nos 12 e nos 18 meses de estudo. A atividade física foi avaliada por questionários e por acelerometria. Para as análises, utilizaram-se o princípio de intenção de tratar e equações de estimativas generalizadas.

**RESULTADOS:**

Após 12 meses, ambos os grupos de intervenção aumentaram os minutos semanais de atividade física no lazer e os escores anuais de exercícios físicos, de lazer e de deslocamento. O grupo das classes de exercícios físicos obteve maior média de escore anual de exercícios físicos em comparação com os outros grupos (p < 0,001). No período pós-intervenção, o grupo de classes de exercícios físicos reduziu o escore anual de exercícios físicos (média: -0,3; IC95% -0,5–-0,1), enquanto o grupo de educação em saúde aumentou este escore (média: 0,2; IC95% 0,1–0,4). Não houve diferenças nos níveis de atividade física mensurados por acelerometria.

**CONCLUSÕES:**

As intervenções foram efetivas para aumentar a prática de atividade física. No entanto, observou-se que a intervenção de educação em saúde foi mais efetiva para a manutenção da prática de atividade física no período pós-intervenção. Recomenda-se a utilização de ambas as intervenções para a promoção da atividade física no Sistema Único de Saúde, de acordo com as realidades locais de profissionais, instalações e objetivos das equipes.

## INTRODUCTION

The Public Health concern with physical inactivity of the world adult population has encouraged the assessment of interventions that encourage the regular practice of physical activity (PA)^10^. In fact, studies conducted in high-income countries have shown that interventions within the context of primary health care have increased the practice of PA in physically inactive adults^19^.

Interventions that implement strategies such as educational actions that enhance autonomy, goal setting, facing of barriers, and guidance for using recreational equipments and available programs in the vicinity of residences, carried out in group meetings and individual counseling, have been shown to be effective to increase PA in adults^11,13^.

Results from health education interventions for the improvement of lifestyle, based on multi-component strategies, are as effective as physical exercise classes to increase or maintain the level of PA in adults^4,18^. Hoehner et al.^12^ have identified that intervention studies based on multi-component strategies are promising in the promotion of physical activity. However, in the context of primary health care in the Latin America, there is little evidence about the effectiveness of these models in the level of PA of adults living in areas with social and economic inequalities^3,12^.

In Brazil, the characteristics of the Unified Health System (SUS) – which operates throughout the country and is based on ensuring free and universal access to health services and actions and reducing social and regional disparities^15^ – and the Family Health Strategy^16^ provide opportunities for the development of interdisciplinary actions to promote large-scale PA, reaching populations residing in regions of low socioeconomic status.

Therefore, the objective of this study was to assess the effects of interventions on health education and exercise classes in the levels of physical activity of adult users of the SUS.

## METHODS

### Design

We have carried out a non-randomized, controlled intervention in primary health care units in the district of Ermelino Matarazzo, located at the eastern side of the city of São Paulo, State of São Paulo, Brazil. This district is among the most socioeconomic vulnerable in the city, taking into account factors such as population growth, income, education level, infant mortality, mortality from external causes, and housing^a^.

### Selection of the Sample

We selected the three primary health care units with Family Health Strategy within the district of Ermelino Matarazzo. The decision on which units would receive interventions or would be the control group was carried out by trial, considering the characteristics and feasibility of each region where they were located, so that the different interventions could be carried out.

According to the inclusion criteria, we selected 157 adults (≥ 18 years) who: did not practice leisure PA in the month preceding the date of the interview or 150 minutes or more of transport-related physical activity in the week before the interview; had no diagnoses of diseases such as diabetes, severe hypertension, cancers, or cognitive diseases that could make them unable to respond to the questionnaires; did not have class III obesity; did not plan to move from the district in two years; and, in the case of women, were not pregnant. The draw of the participants was conducted according to the registration of users in the units.

Participants were placed in three groups: health education intervention (n = 54), exercise class intervention (n = 54), and control (n = 49).

All the details of the process of selection of units and sample of users can be obtained from the publication of Salvador et al.^24^

### Interventions

Users placed in the health education group participated in 16 thematic meetings on healthy lifestyle. This intervention aimed to develop autonomy for the practice of PA, as well as the adoption of healthy eating and stress control. The meetings were conducted in groups of eight to 13 persons and lasted for 120 minutes each, and the last 20 minutes were intended for experiences of PA. If the individual missed the session, an individual intervention was made with a telephone call with an average duration of 20 minutes. This intervention was conducted by the research team composed of physical education professionals, nutritionist, physician, and psychologist and was based on social cognitive theory^2^ and the ecological model for the promotion of PA^23^. Thematic meetings were held in the health unit and in an association of neighborhood residents.

Users of the exercise class group participated in three weekly sessions of cardiorespiratory, muscle strength, and flexibility exercises carried out in groups of 10 to 15 persons, conducted and supervised by a physical education professional. Activities of walking, running, stretching, and muscle strengthening circuits were carried out. Every session lasted for 60 minutes and the activities were planned according to the recommendations of the American College of Sports Medicine^7^. The intensity and volume of physical exercises were adjusted periodically over the 12 months. This intervention was conducted at the Escola de Artes, Ciências e Humanidades of the Universidade de São Paulo.

All interventions lasted for 12 months (from March/April 2011 to March/April 2012), followed by six months of follow-up, amounting to 18 months of study. A control group, which received no intervention, was used for the comparison with groups. More details about the interventions can be obtained from the publication of Salvador et al.^25^

### Assessments

The assessments of PA using questionnaires were made in three periods: 1) at baseline, 2) twelve months after the starting of interventions, and 3) six months after the ending of interventions.

Measurements by accelerometry were made only in periods two and three of the study. The outcomes of this study were:

1. minutes per week in leisure and transport-related PA: assessed by the modules of leisure and transport-related PA of the long and adapted version of the International Physical Activity Questionnaire (IPAQ)^14^. The IPAQ investigates the activities carried out in the seven days before the interview. The daily duration of walks and moderate and vigorous activities was multiplied by the weekly frequency. Vigorous activities were multiplied by two. Transport-related PA was obtained by the sum of the weekly minutes of walking or cycling, calculated by multiplying the weekly frequency by the daily duration.
2. annual scores of physical activities: obtained by the Baecke questionnaire^5^. This tool investigates the usual activities carried out in the 12 months before the interview and generates scores in a numeral scale. In this study, we used the annual scores of physical exercises and leisure and transport activities, in addition to the sum of those two scores.
3. daily minutes in moderate and vigorous activities: assessed using an ActiGraph accelerometer, models GT1M and GT3X+. Participants used the accelerometer for eight consecutive days in the waist, removing the equipment only to sleep and take a shower. The data were collected with *epoch* of 60 seconds and treated in the software ActiLife 6.8 using the algorithm *daily*, considering 60 consecutive zeros as invalid hour. Valid days were defined as having at least 10 hours of use. Only participants with data on four or more valid days, being one in the weekend, were included in the analyses. The cut-off points of Freedson et al.^6^defined the moderate and vigorous activities. The daily minutes in moderate and vigorous activities were obtained from the division of total minutes in these activities by the amount of valid days.

We assessed the validity of the IPAQ and Baecke questionnaires by comparing with the accelerometry in a sub-sample of adults in this study^8^. Correlation coefficients were obtained between 0.34 and 0.36 in the comparison between the methods. Adults who reached at least 150 minutes per week in leisure or transport-related physical activities estimated by the IPAQ and who were in the highest tertiles of the scores of PA for the Baecke questionnaires also presented higher averages of weekly minutes in moderate and vigorous activities measured by accelerometry.

The questionnaires on PA were applied by trained and independent interviewers, who did not participate in the interventions. At the end of the application of questionnaires, in the 12- and 18-month assessment, the interviewers gave and guided the participants regarding the use of accelerometers.

The variables gender, age group (18 to 39 years, ≥ 40 years), education level (≤ 8 years, ≥ 9 years), work (yes, no), race (white, non-white), and marital status (with or without partner) were assessed using a questionnaire. Weight and height, measured directly, were used to calculate body mass index (BMI) and we considered as normal weight if BMI < 25 kg/m^2^ and as overweight if BMI ≥ 25 kg/m^2^. Social and demographic variables and BMI were collected at the beginning and in the 12 and 18 months of study; however, the frequencies shown are related only to the initial assessment.

More details about the assessment tools can be obtained from the publication of Salvador et al.^25^

### Analysis of Data

The variables were analyzed according to the intention-to-treat principle. For this end, in the case of missing data, we repeated the most recently data collected from that person as the method of data imputation^17^.

The Chi-square test was conducted to compare the categorical variables of gender, age group, education level, race, marital status, and BMI of the groups at baseline.

The effect of the groups on the level of PA between the moments of assessment was analyzed by generalized estimating equations. For the annual scores of PA, we used the normal distribution function and the identity link function. For the weekly and daily variables of PA, we used the inverse Gaussian distribution function and the identity link function. For these variables, we added one minute per week or one minute per day to all values to prevent null values (values necessarily must be > 0 in an inverse Gaussian distribution). In all cases, we used an unstructured correlation matrix, so that each variance and covariance could be estimated independently, and the Huber-White estimator to account for the possible heteroscedasticity resulting from the calculation of standard errors^26^. For each outcome, we estimated the effect of belonging to a certain group of intervention, the time elapsed since the beginning of the intervention (baseline and 12 and 18 months of study), and the interaction of these two factors, to assess possible differences in the temporal trends of PA between groups. For outcomes involving the practice of PA as transport, the model was adjusted for race, because initial differences were identified in the groups in this type of PA according to this variable (data not presented).

The Mann-Whitney test was also used to compare the initial pattern of PA of the participants assessed and not assessed in 12 and 18 months (Table 1). In all analyses, we considered a descriptive level of 5%. The analyses were carried out using the statistical program SPSS, version 22.0.

**Table 1:** Comparison of the averages (standard deviations) of physical activity, at baseline, of adult users of the Brazilian Unified Health System and attended by the Family Health Strategy, Ermelino Matarazzo, São Paulo, State of São Paulo, Brazil, assessed and not assessed in 2012.

Variable	12 months		18 months		p*_12_	p*_18_
	
	Assessed	Not assessed	Assessed	Not assessed		
	
	n = 114	n = 43	n = 110	n = 47		
Transport-related physical activity (min/week)	41.38 (42.88)	50.91 (46.26)	47.19 (44.15)	36.49 (42.80)	0.25	0.15
Annual score of physical exercises	2.00 (0.51)	1.91 (0.62)	2.01 (0.54)	1.89 (0.54)	0.30	0.17
Annual score of leisure and transport activities	2.23 (0.53)	2.27 (0.55)	2.25 (0.56)	2.22 (0.47)	0.85	0.67
Sum of annual scores of physical exercises and leisure and transport activities	4.25 (0.78)	4.18 (0.96)	4.27 (0.85)	4.12 (0.77)	0.56	0.25

* p-values corresponding to the Mann-Whitney test.

### Ethical Aspects

This study was approved by the Research Ethics Committee of the Municipal Health Department of São Paulo (Protocol 0072.0.162.000-10) and by the Research Ethics Committee of the Faculdade de Saúde Pública of the Universidade de São Paulo (Protocol 01773412.2.0000.5421). The study was registered on the database ClinicalTrials.gov (Identifier NCT01330836). After the end of the study, the control group received an intervention by telephone that summed up the topics discussed in the health education intervention and the persons of the group were invited to participate in a university extension program which brought together the activities developed in the two interventions assessed in this study.

## RESULTS

Most adults in the study were female, aged between 18 and 39 years, with partner, with at least nine complete years of school, and worked (Table 2). There was a higher proportion of non-white persons and they practiced more minutes per week of transport-related PA compared to white persons (p = 0.03) (data not shown).

**Table 2:** Absolute numbers and frequencies (%) of the social and demographic variables and the nutritional status of the total of persons and stratified by study groups for adult users of the Brazilian Unified Health System and attended by the Family Health Strategy. São Paulo, State of São Paulo, Brazil.

Variable	Total		Physical Exercise		Education		Control		p*
			
	n = 157		n = 54		n = 54		n = 49		
			
	n	%	n	%	n	%	n	%	
Gender									
Male	50	31.8	19	35.2	16	29.6	15	30.6	0.81
Female	107	68.2	35	64.8	38	70.4	34	69.4	
Age group (years)									
18–39	100	63.7	40	74.1	34	63.0	26	53.1	0.09
≥ 40	57	36.3	14	25.9	20	37.0	23	46.9	
Education level (years)									
≥ 8	71	45.2	28	51.9	25	46.3	18	26.7	0.30
≥ 9	86	54.8	26	48.1	29	53.7	31	63.3	
Work									
Yes	96	61.1	35	64.8	34	63.0	27	55.1	0.57
No	61	38.9	19	35.2	20	37.0	22	44.9	
Race									
White	58	36.9	14	25.9	19	35.2	25	51.0	0.03
Non-white	99	63.1	40	74.1	35	64.8	24	49.0	
Marital status									
With partner	101	64.3	40	74.1	33	61.1	28	57.1	0.17
No partner	56	35.7	14	25.9	21	38.9	21	42.9	
BMI									
< 25 kg/m^2^	77	49.0	32	59.3	22	40.7	23	46.9	0.15
≥ 25 kg/m^2^	80	51.0	22	40.7	32	59.3	26	53.1	

BMI: body mass index

* p-values calculated by the Chi-square test.

We reassessed 72.6% of the participants using questionnaires at 12 months and 70.1% of the participants at 18 months (Figure). We were able to assess 51.5% of the participants by accelerometry at 12 months and 28% at 18 months. There was no statistically significant difference between the reassessed and non-assessed participants at 12 and 18 months regarding their level of PA assessed by questionnaires at baseline (Table 1).

**Figure:**
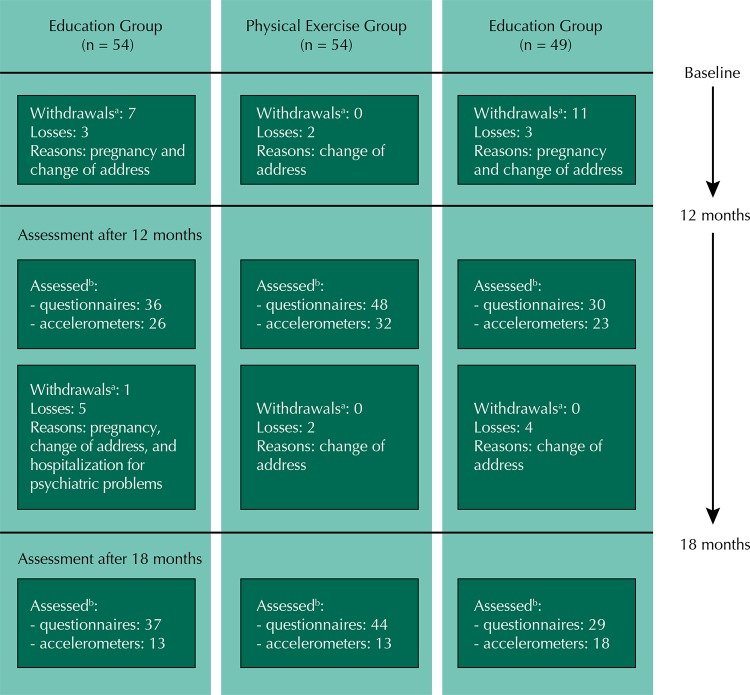
Flowchart of participation according to study group.

^a^ Adults who reported that they were quitting the study.

^b^ Adults who completed the questionnaires or provided valid data for at least four days of use of the accelerometer.

The intervention groups significantly increased the average weekly practice of leisure PA from baseline to 12 months of study (Table 3). The three groups increased the weekly minutes of transport-related PA and the sum of the minutes of leisure or transport-related PA at 12 months when compared to the beginning of the study. In the period after interventions, only the exercise group reduced the weekly practice of transport-related PA and the sum of the minutes of leisure or transport-related PA.

**Table 3:** Intra-group differences of averages and 95% confidence intervals estimateda for the levels of physical activity of adult users of the Brazilian Unified Health System, attended by the Family Health Strategy. São Paulo, State of São Paulo, Brazil.

Groups	Baseline × 12 months^b^		12 months × 18 months^c^	
	
	Average	95%CI	Average	95%CI
Leisure time physical activity (min/week)				
Exercise	53	20–86^e^	-31	-64–3
Education	35	3–67^e^	6	-13–25
Control	28	-1–56	-9	-49–30
Transport-related physical activity (min/week)^d^				
Physical exercise	90	42–138^e^	-56	-102– -9^e^
Education	105	49–161^e^	-7	-40–54
Control	45	13–77^e^	22	-6–50
Leisure and transport-related physical activity (min/week)^d^				
Exercise	141	91–191^e^	-84	-140– -28^e^
Education	141	72–210^e^	-1	-48–46
Control	73	27–118^e^	13	-38–63
Annual score of physical exercises				
Exercise	0.6	0.3–0.8^e^	-0.3	-0.5– -0.1^e^
Education	0.1	-0.1–0.3	0.2	0.1–0.4^e^
Control	-0.1	-0.2–0.1	0.1	-0.1–0.3
Annual score of leisure and transport activities^d^				
Exercise	0.3	0.1–0.5^e^	-0.2	-0.4–0.1
Education	0.3	0.1–0.4^e^	0.1	-0.1–0.2
Control	0.2	-0.1–0.3	0.1	-0.2–0.2
Annual scores of physical exercises and leisure and transport activities^d^				
Exercise	0.8	0.5–1.2^e^	-0.5	-0.8– -0.1^e^
Education	0.4	0.2–0.7^e^	0.3	-0.1–0.6
Control	0.1	-0.1–0.3	0.1	-0.2–0.4
Moderate to vigorous activities (min/day)^3^				
Exercise	-	-	2	-1–5
Education	-	-	3	-2–9
Control	-	-	1	-2–4

min/week: minutes of practice per week; min/day: minutes of practice per day

^a^ Values estimated by generalized estimating equations.

^b^ Comparison of initial values with those of 12 months of study.

^c^ Comparison of the values for 12 months with those of 18 months of study.

^d^ Analysis adjusted for race.

^e^ Significant difference determined by Bonferroni post hoc test.

The exercise class group increased the annual exercise score from baseline to 12 months, but decreased it from 12 to 18 months. On the other hand, the health education group increased the average of this score from 12 to 18 months. Both intervention groups significantly increased the average annual scores of leisure and transport-related PA and the sum of the scores of physical exercise and leisure and transport-related PA from baseline to 12 months, but the exercise class group reduced this sum significantly in the period from 12 to 18 months.

There were no differences in moderate and vigorous physical activities measured by accelerometers.

Table 4 presents the estimated averages of the study groups and comparisons of these values according to group, time, and time and group interaction.

**Table 4:** Averages (95%CI) estimateda for the levels of physical activity of adult users of the Brazilian Unified Health System, attended by the Family Health strategy. São Paulo, State of São Paulo, Brazil.

Groups	Baseline	12 months^b^	18 months^c^	p _group_^e,f^	p _time_^e,g^	p _interaction_^e,h^
Leisure time physical activity (min/week)						
Exercise	-	54 (27–81)	23 (7–39)	0.409	< 0.001	0.194
Education	-	26 (10–62)	42 (14–69)			
Control	-	29 (6–52)	19 (-2–41)			
Transport-related physical activity (min/week)^d^						
Exercise	50 (39–61)	140 (102–179)	85 (67–103)	0.655	< 0.001	< 0.001
Education	32 (20–43)	137 (88–185)	130 (95–164)			
Control	47 (35–60)	93 (68–118)	114 (85–144)			
Leisure and transport-related physical activity (min/week)^d^						
Exercise	50 (39–61)	191 (150–232)^i^	107 (82–133)	0.358	< 0.001	0.002
Education	32 (20–43)	173 (113–233)	172 (123–232)			
Control	47 (35–60)	120 (84–156)	132 (98–167)			
Annual score of physical exercises						
Exercise	2.0 (1.9–2.2)	2.6 (2.4–2.8)^i,j^	2.3 (2.2–2.5)^i^	0.003	< 0.001	< 0.001
Education	1.9 (1.8–2.0)	2.1 (1.9–2.2)	2.3 (2.1–2.5)			
Control	2.0 (1.8–2.2)	1.9 (1.7–2.1)	2.0 (1.8–2.2)			
Annual score of leisure and transport activities^d^						
Exercise	2.3 (2.2–2.5)^j^	2.6 (2.5–2.8)	2.4 (2.3–2.6)	0.139	< 0.001	0.097
Education	2.1 (2.0–2.2)	2.4 (2.2–2.5)	2.4 (2.3–2.6)			
Control	2.2 (2.1–2.4)	2.4 (2.2–2.6)	2.4 (2.2–2.6)			
Sum of annual scores of physical exercises and leisure and transport activities^d^						
Exercise	4.4 (4.1–4.6)	5.2 (4.9–5.5)^i,j^	4.7 (4.5–5.0)	0.006	< 0.001	< 0.001
Education	4.0 (3.8–4.2)	4.4 (4.1–4.7)	4.7 (4.4–5.0)			
Control	4.3 (4.0–4.5)	4.4 (4.1–4.6)	4.4 (4.1–4.7)			
Moderate and vigorous activities (min/day)^d^						
Exercise	-	32 (25–39)	34 (26–42)	0.766	0.071	0.712
Education	-	28 (20–36)	31 (22–41)			
Control	-	30 (24–36)	31 (23–36)			

min/week: minutes of practice per week; min/day: minutes of practice per day

^a^ Values estimated by generalized estimating equations.

^b^ Ending of the intervention period.

^c^ Ending of the follow-up period.

^d^ Analysis adjusted for race.

^e^ p-values determined by generalized estimating equations.

^f^ Comparison of the averages presented by the study groups, independent of time.

^g^ Comparison of the general averages between assessment periods, independent of study groups.

^h^ Values corresponding to the time*group interaction.

^i^ Statistically significant difference between the groups of supervised physical exercise and control.

^j^ Significant difference between intervention groups.

The averages of transport-related PA of the groups were different, and the exercise class group showed the highest average at baseline and 12 months, and the health education group showed the highest average at 18 months. Compared to the control group, the exercise class group showed the highest average of minutes per week of leisure and transport-related physical activities at 12 months.

The exercise class group showed the highest average scores of physical exercises at 12 months when compared with the other two groups. However, at 18 months, this difference remained only in the comparison with the control group. The annual score of leisure and transport activities was different only in the beginning of the study, with a higher average for the exercise class group compared to the health education group. In the sum of the annual scores of physical exercise and leisure and displacement activities, the exercise class group obtained the highest average at 12 months compared to the other two groups; however, there were no differences in the period of 18 months.

There were no differences in moderate and vigorous activities assessed by accelerometry according to group, time, and time and group interaction.

## DISCUSSION

The results of this article showed that both the intervention based on health education and the intervention based on physical exercise classes were effective to increase the practice of PA in adult users of the SUS who live in a region of low socioeconomic status. However, we observed that only the health education intervention was effective in maintaining the level of PA in the six months of follow-up after the intervention.

The results found in this study were similar to that of Dunn et al.^4^, carried out with 235 healthy adult Americans, and Opdenacker et al.^18^, carried out with 186 older adults Belgians. Both research studies have found that interventions based on lifestyle improvement and development of autonomy are as effective as traditional exercise classes for the improvement of the practice of PA.

For the six months of follow-up after interventions, the results obtained in this study were similar to those found by Opdenacker et al.^18^, who have used 12 months of follow-up after the end of interventions showing that the most favorable results were found for persons who participated in the health education intervention.

We highlight that the significant results obtained with the exercise class intervention can be related to the sessions offered for this group, not necessarily to the addition of new physical activity practices, and why we chose not to remove them from the analyses.

In this sense, Zorzetto^27^ has compared two interventions of physical exercise classes, one with only three weekly sessions and the other with two weekly sessions and guidelines on PA and healthy habits for 82 adult women, in primary health care units of Rio Claro, State of São Paulo, Brazil. The results of the analyses considering the minutes intended for the practice of activities offered by the interventions showed that both groups significantly increased the practice of leisure time PA in 12 months, with better outcome among the women who participated in the three weekly sessions of physical exercises^27^. However, when the time offered in the interventions was removed from the analyses, no significant difference was observed^27^.

The strategies used to intervene in the health education group involved several components, such as individual counseling by telephone, setting of individual goals, and, for the groups, face-to-face guidance for the overcoming of barriers, use of printed materials, and experience sessions on exercises, including cognitive, behavioral, and social aspects. Results of systematic reviews show that interventions that use multi-component strategies, such as group meetings, use of educational printed materials, and individual counseling, have significantly increased the levels of PA in adults^10-13,19^.

Interventions such as health education use techniques that assist in the improvement of autonomy and individual and community empowerment for behavior change, such as the use of existing public spaces, self-care, and respect for the individual, working complex issues such as security and barriers for the practice of PA, and the search for problem solving with a participatory, interdisciplinary, and intersectoral perspective. These interventions are in accordance with what is advocated for actions of Support Centers for Family Health in the territories^16^.

The activities for this model do not require specific equipment installed directly in the health units, which can include the promotion of other behaviors together with the practice of PA and be mediated by professionals from different areas of knowledge, in accordance with the subject addressed. In the case of Brazil, it is important to note that actions, related to education, information, and communication, are being used in programs for the promotion of physical activity^1^.

However, we highlight that physical exercise classes are commonly used in health units in Brazil and they can complement the actions of the health education groups, especially with physical education professionals present in the Support Centers for Family Health. For example, a study published in 2014 showed that walking groups are the most used strategies of health promotion in primary health care units in Brazil and the professionals of physical education are the main players responsible for the management of the actions for the promotion of PA^9^.

The main limitations of this study are related to the methods of assessment and the allocation of health units. Significant results came from data collected by questionnaires. The self-reported assessment of PA, despite allowing the identification of practices in different domains, may have been overestimated^21^. The differences found by the questionnaires were not confirmed in the direct measures from accelerometry, because there were problems in the data collections, such as the absence of this measure at the beginning of the study and the losses that occurred in the assessments of accelerometers at 12 and 18 months. The absence of randomization may also have created problems in the comparison of groups^20^. For example, at the beginning of the study, the groups were different in relation to transport-related PA according to race. It is also important to note that interventions developed in this study offered activities at night and on Saturdays, days and time that are outside the regular schedule of primary health care units.

To minimize some of these issues, the interviews to assess PA, at 12 and 18 months, were conducted by interviewers other than the professionals who carried out the interventions in the groups. In addition, all questionnaires used in this research obtained evidence of validity in the comparison with accelerometry^8^. Regarding the choice of the units, we did it taking into account the structural conditions of each coverage area of the health units to accomplish each type of intervention^22^.

From the results of this study, we recommend the use of both interventions to promote physical activity in the Brazilian Unified Health System, according to the local reality of professionals, facilities, and team objectives. Additionally, the municipal administration of health services must reflect on working hours of the health care teams, since some of the adult users of the SUS who live in areas of low socioeconomic status may have problems to participate in interventions in the current times of the primary health care units.

## Footnotes

a Prefeitura da Cidade de São Paulo. Atlas Ambiental do Município de São Paulo. Available from: http://atlasambiental.prefeitura.sp.gov.br/conteudo/socioeconomia/socioeco_13.jpg
